# Supplementary material for: Predicted action-effects shape action representation through pre-activation of alpha oscillations
Source: Commun Biol. 2025 Feb 22;8:275. doi: 10.1038/s42003-025-07750-4 (PMC11846963; doi:10.1038/s42003-025-07750-4)
Supplement: Supplementary file 2 — Supplementary Material [file 42003_2025_7750_MOESM2_ESM.pdf]

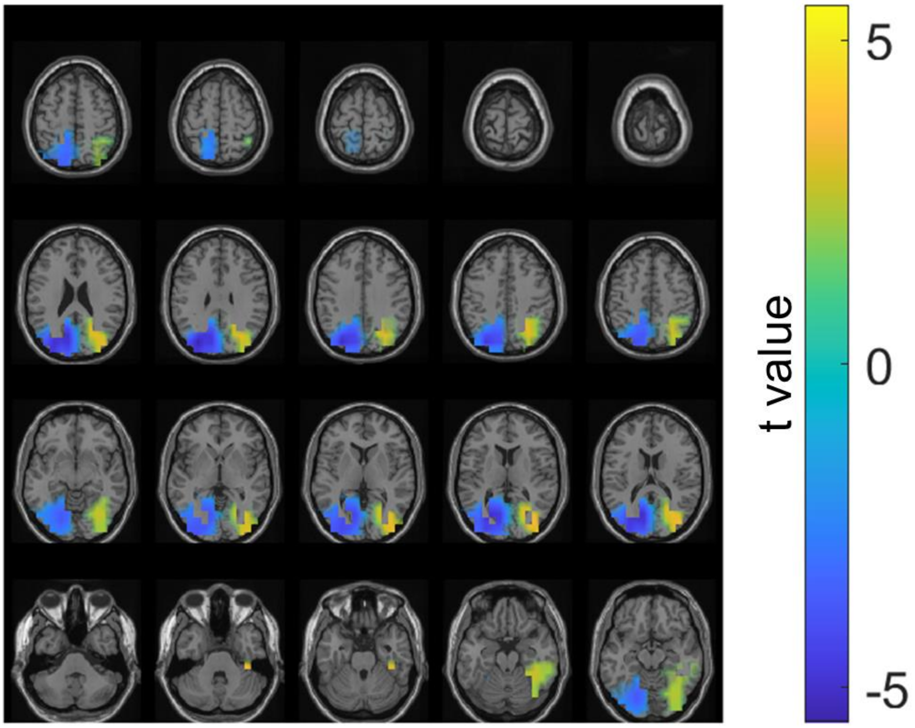

Supplementary Figure 1 Source localisation results of the SSVEP response. The SSVEP response was strongest in the visual cortex, including bilateral calcarine fissure, lingual gyrus, fusiform gyrus, cuneus, superior occipital gyrus, middle occipital gyrus and inferior occipital gyrus. Other activated brain areas include cingulate, superior parietal gyrus, inferior parietal gyrus, angular gyrus, precuneus, middle temporal gyrus, and inferior temporal gyrus. See also Fig 4c in the main text.

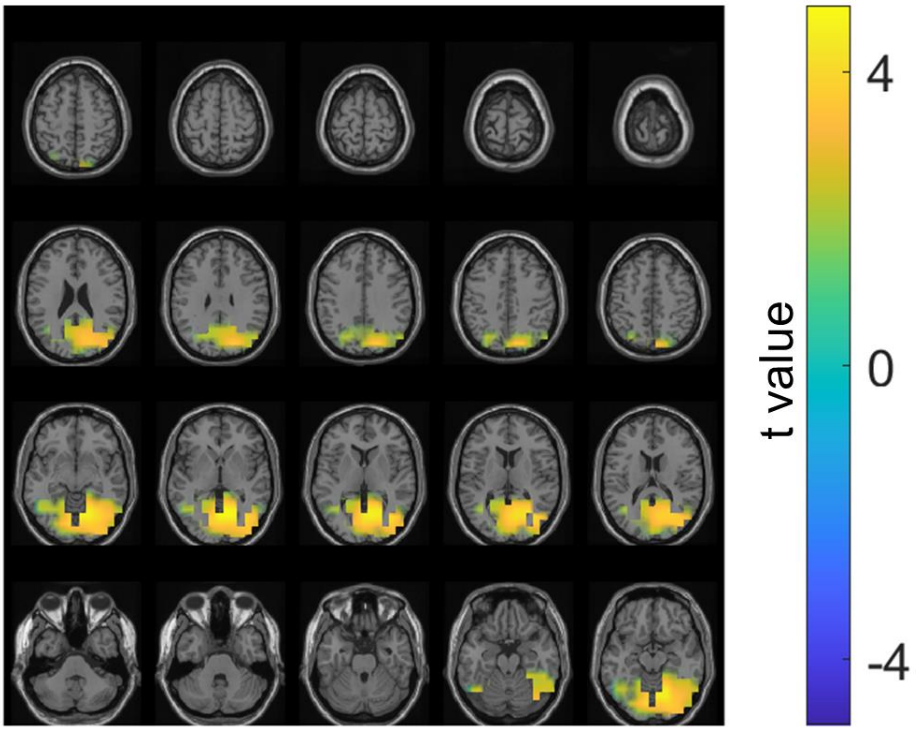

Supplementary Figure 2 Source localisation results of the alpha power difference between the instrumental and baseline conditions in the time window [-3000 -2000] ms. Stronger alpha power in the instrumental condition was mainly found in the visual cortex, including bilateral calcarine fissure, lingual gyrus, fusiform gyrus, cuneus, superior occipital gyrus, middle occipital gyrus and inferior occipital gyrus. Other significant brain areas include bilateral superior parietal gyrus, precuneus, middle temporal gyrus, inferior temporal gyrus, left posterior cingulate gyrus, right parahippocampal gyrus, and right angular gyrus. See also Fig 5b in the main text.

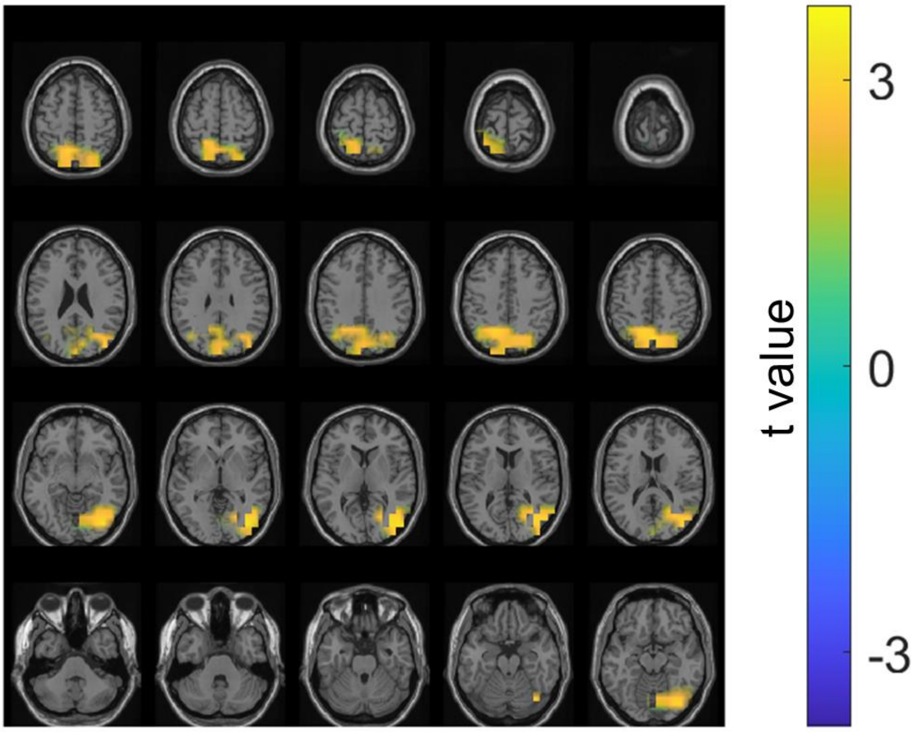

Supplementary Figure 3 Source localisation results of the alpha power difference between the instrumental and baseline conditions in the time window [-1800 -800] ms. Stronger alpha power in the instrumental condition was mainly found in the visual cortex, including right calcarine fissure, right lingual gyrus, right fusiform gyrus, right inferior occipital gyrus, bilateral cuneus, superior occipital gyrus, and middle occipital gyrus. Other significant brain areas include bilateral superior parietal gyrus, precuneus, left inferior parietal gyrus, left postcentral gyrus, left angular gyrus, right middle temporal gyrus, and right inferior temporal gyrus. See also Fig 5b in the main text.
