# Supplementary material for: Predicted action-effects shape action representation through pre-activation of alpha oscillations
Source: Commun Biol. 2025 Feb 22;8:275. doi: 10.1038/s42003-025-07750-4 (PMC11846963; doi:10.1038/s42003-025-07750-4)
Supplement: Supplementary file 3 — Reporting Summary [file 42003_2025_7750_MOESM3_ESM.pdf]

nature portfolio

Corresponding author(s): Liyu Cao

Last updated by author(s): Feb 11, 2025

Reporting Summary

Nature Portfolio wishes to improve the reproducibility of the work that we publish. This form provides structure for consistency and transparency in reporting. For further information on Nature Portfolio policies, see our Editorial Policies and the Editorial Policy Checklist.

Statistics

For all statistical analyses, confirm that the following items are present in the figure legend, table legend, main text, or Methods section.

n/a	Confirmed	
☐	☒	The exact sample size (*n*) for each experimental group/condition, given as a discrete number and unit of measurement
☐	☒	A statement on whether measurements were taken from distinct samples or whether the same sample was measured repeatedly
☐	☒	The statistical test(s) used AND whether they are one- or two-sided<br>*Only common tests should be described solely by name; describe more complex techniques in the Methods section.*
☐	☒	A description of all covariates tested
☐	☒	A description of any assumptions or corrections, such as tests of normality and adjustment for multiple comparisons
☐	☒	A full description of the statistical parameters including central tendency (e.g. means) or other basic estimates (e.g. regression coefficient) AND variation (e.g. standard deviation) or associated estimates of uncertainty (e.g. confidence intervals)
☐	☒	For null hypothesis testing, the test statistic (e.g. *F*, *t*, *r*) with confidence intervals, effect sizes, degrees of freedom and *P* value noted<br>*Give P values as exact values whenever suitable.*
☒	☐	For Bayesian analysis, information on the choice of priors and Markov chain Monte Carlo settings
☐	☒	For hierarchical and complex designs, identification of the appropriate level for tests and full reporting of outcomes
☐	☒	Estimates of effect sizes (e.g. Cohen's *d*, Pearson's *r*), indicating how they were calculated

Our web collection on statistics for biologists contains articles on many of the points above.

Software and code

Policy information about availability of computer code

Data collection	For the behavioural experiments, we used:<br>Matlab (version: 9.6.0.1472908 (R2019a) Update 9) and Psychtoolbox (version: 3.0.18) for stimulus presentation;<br>Lab Streaming Layer (version: 1.16.2) for the data collection.<br>For the EEG experiment, we used:<br>Matlab (version: 9.13.0.2049777 (R2022b)) and Psychtoolbox (version: 3.0.19.7) for stimulus presentation;<br>ActiView (version: 8.09) for the data collection.
Data analysis	We used Matlab (version: 9.10.0.1684407 (R2021a) Update 3), Fieldtrip toolbox (version: 20230302), Lab Streaming Layer (version: 1.10), and teg_RMA (version: 1.0).

For manuscripts utilizing custom algorithms or software that are central to the research but not yet described in published literature, software must be made available to editors and reviewers. We strongly encourage code deposition in a community repository (e.g. GitHub). See the Nature Portfolio guidelines for submitting code & software for further information.

Data

Policy information about availability of data

All manuscripts must include a data availability statement. This statement should provide the following information, where applicable:

- Accession codes, unique identifiers, or web links for publicly available datasets
- A description of any restrictions on data availability
- For clinical datasets or third party data, please ensure that the statement adheres to our policy

The raw data reported in this manuscript have been uploaded to Open Science Framework for free access (https://doi.org/10.17605/OSF.IO/QVX8W)

Research involving human participants, their data, or biological material

Policy information about studies with human participants or human data. See also policy information about sex, gender (identity/presentation), and sexual orientation and race, ethnicity and racism.

Reporting on sex and gender	We have collected data from 120 individuals in total in the 4 experiments reported in the manuscript. Of the 120 individuals, 54 are female and 66 are male. Sex was not considered in the study design. Sex was determined based on self-reporting. We did not perform any sex-based analyses, as we did not have a prior hypothesis on the possible role of sex in the studied effect.
Reporting on race, ethnicity, or other socially relevant groupings	We did not use any socially constructed or socially relevant categorization variables in the current study.
Population characteristics	In experiment 1, the 30 participants have a mean age of 21.7 (SD = 2.8).<br>In experiment 2, the 30 participants have a mean age of 22.3 (SD = 2.6).<br>In experiment 3, the 30 participants have a mean age of 23.0 (SD = 3.2).<br>In experiment 4, the 30 participants have a mean age of 23.5 (SD = 3.9).<br>All participants are healthy.
Recruitment	Participants were recruited from a local participant pool. We are not aware of any potential self-selection bias or other biases that may be present or have an impact on the results.
Ethics oversight	The Ethics Committee of Department of Psychology and Behavioural Sciences, Zhejiang University

Note that full information on the approval of the study protocol must also be provided in the manuscript.

Field-specific reporting

Please select the one below that is the best fit for your research. If you are not sure, read the appropriate sections before making your selection.

☒ Life sciences ☐ Behavioural & social sciences ☐ Ecological, evolutionary & environmental sciences

For a reference copy of the document with all sections, see nature.com/documents/nr-reporting-summary-flat.pdf

Life sciences study design

All studies must disclose on these points even when the disclosure is negative.

Sample size	In the first experiment, there were 30 participants. The sample size was determined based on our prior experience in similar studies. With 30 participants, the smallest effect size that can be detected with a statistical power of 0.9 at the standard 0.05 alpha error probability (one tailed) using paried sample t test is 0.55 (calculated with GPower version 3.1.9.7; Faul, et al.). The action binding effect studied here is estimated to have an effect size of about 0.70. For the critical effect of an attention distribution difference between different conditions, the smallest effect size (partial eta squared) of an interaction effect with within-participants 2 by 8 ANOVA that can be detected with a statistical power of 0.9 at the standard 0.05 alpha error probability is 0.09 (calculated with MorePower version 6.0.1).<br>The other 3 experiments addressed the same question as the first experiment from different angles. Therefore, we kept the sample size the same (i.e. 30) for all experiments in the current study.
Data exclusions	In experiment 1, 5 participants were excluded.<br>In experiment 2, 5 participants were excluded.<br>In experiment 3, 3 participants were excluded.<br>Experiments 1-3 are behavioural experiments. Excluded participants have behavioural responses that are identified as outliers, and therefore unreliable, using the MAD-median rule. We have always consistently used the MAD-median rule to exclude participants, as has been performed in several already published papers studying the same effect.<br>In experiment 4, 3 participants were excluded. The excluded participants do not have enough trials for the data analysis due to missing responses or extremely noisy EEG data.<br>The data exclusion criteria used in the current study are very standard in the research field. Therefore, we consider the exclusion criteria pre-established.

Replication	The action binding effect was shown several times in the current study (experiments 1,3, and 4). Therefore, it is highly replicable. The attention shift effect was shown in both behavioural (experiment 1) and EEG experiments (experiment 4). It is also replicable. The crutial alpha effect was found in both between-condition comparison and cross-participant correlation analyses. Therefore, it is a highly reliable effect.
Randomization	We have within-participants design for all the experiments reported in the study. Therefore, the randomization issue is not relevant here.
Blinding	Blinding was not relevant to the current study.<br>The current study always uses a within-participants design. Each participant was tested in both baseline and experimental conditions. During the experiment, the experimenter was around for the instructions and practice trials, so the experimenter knew the testing condition. However, during the formal data collection, the participant sat alone in the testing booth without the experimenter around.<br>For the data analysis, the two conditions (baseline and experimental) were pre-processed together with the same criteria. Binding or not blinding does not have any influence on the results.

Reporting for specific materials, systems and methods

We require information from authors about some types of materials, experimental systems and methods used in many studies. Here, indicate whether each material, system or method listed is relevant to your study. If you are not sure if a list item applies to your research, read the appropriate section before selecting a response.

Materials & experimental systems

n/a	Involved in the study
☒	☐ Antibodies
☒	☐ Eukaryotic cell lines
☒	☐ Palaeontology and archaeology
☒	☐ Animals and other organisms
☒	☐ Clinical data
☒	☐ Dual use research of concern
☒	☐ Plants

Methods

n/a	Involved in the study
☒	☐ ChIP-seq
☒	☐ Flow cytometry
☒	☐ MRI-based neuroimaging

Plants

Seed stocks	*Report on the source of all seed stocks or other plant material used. If applicable, state the seed stock centre and catalogue number. If plant specimens were collected from the field, describe the collection location, date and sampling procedures.*
Novel plant genotypes	*Describe the methods by which all novel plant genotypes were produced. This includes those generated by transgenic approaches, gene editing, chemical/radiation-based mutagenesis and hybridization. For transgenic lines, describe the transformation method, the number of independent lines analyzed and the generation upon which experiments were performed. For gene-edited lines, describe the editor used, the endogenous sequence targeted for editing, the targeting guide RNA sequence (if applicable) and how the editor was applied.*
Authentication	*Describe any authentication procedures for each seed stock used or novel genotype generated. Describe any experiments used to assess the effect of a mutation and, where applicable, how potential secondary effects (e.g. second site T-DNA insertions, mosiacism, off-target gene editing) were examined.*
